# Dangers and Reactions of Radium Treatment

**Published:** 1914-03-21

**Authors:** 


### Dangers and Reactions of Radium Treatment.

Like the *x*-rays, the radiations of radium may be a source of danger both to patients and operators. In the early days of *x*-ray treatment a good many cases of burning took place, before the dangers were appreciated and plans to avert them adopted. Those who were repeatedly exposed to the rays were ultimately affected by intractable cancers; some lost limbs only; others life as well. Hitherto tragedies of this sort have not marked the use of radium; but cases have been reported which may quite possibly turn out to be disastrous in the end. At any rate, no precaution should be omitted by radium workers to guard against these risks.

An erythema of the skin, amounting even occasionally to a burn, is not by any means rare on surfaces that have been exposed to radio-activity. In rare cases ulceration may take place, and sloughing has also been known to occur when tubes of radium or its emanation have been implanted into the substance of a tumour. In cases where radium has been used near the peritoneal cavity, fæcal fistulæ have been reported in occasional cases, from the action of the rays upon adherent coils of intestine. Thrombosis is another danger which attends the implantation of radium into situations which are close to large blood-vessels, such as the jugular and femoral veins. Fatal embolism may ensue. Hæmorrhage is another complication which sometimes follows radium treatment. Another untoward result is the causation of painful and persistent local reaction, which is sometimes troublesome after the use of radium in the rectum and vagina. Many of these dangers can be minimised by careful screening of the dose of radium used; but there are almost as many opinions on the best sorts of screens as upon the optimum dosage for various diseases.

The truth of the matter is that the exact effects of the $\alpha$, $\beta$, and $\gamma$ rays upon different tissues and organs, and their powers of penetration, are not by any means fully worked out. Individual susceptibilities also play an important part; for some patients will evince much more marked reaction than others to radio-active exposures which are identical in every respect. This brings us naturally to the consideration of the theoretical basis of radium treatment.

It is easy enough to prove that radiations can and do influence the tissues of the human body. Indeed, the facts of *x*-ray therapy (and of *x*-ray accidents) are sufficient evidence of this. There is also abundance of experimental proof that the radiations from radium have the same powers. One of the great obstacles in getting down to the first principles of radio-therapeutics is the difficulty of getting the $\alpha$, the $\beta$, or the $\gamma$ rays, respectively, pure and unmixed with each other. This difficulty has been partially, but not wholly, overcome.

Radium emanation, it is true, gives out many more of the $\alpha$ rays than of the $\beta$ and $\gamma$ rays; but it is not safe to conclude from this that the effects of emanation treatment are mainly the result of $\alpha$ radiation. There is, in fact, still a good deal of uncertainty as to the precise effects of these $\alpha$-rays.

The $\beta$-rays affect all organs and tissues of the body to a greater or less degree if they are applied in sufficient quantity and for a sufficient length of time. Some observers state that the blood vessels, others that the nervous tissues are most affected. Mucous membranes are much more sensitive to $\beta$-rays than unbroken skin. Some organs react in different ways from others, and the dosages required to destroy some types of cell are much greater than those which will have the same effect upon other types. In general, it may be said that small doses are stimulating, large doses destructive in action. But it must be remembered that a dose strong enough to destroy one cell may suffice merely to stimulate another. It has further to be noted that the amount of radiation which reaches a part or organ near the source of the rays will be greater, and may be very much greater, than that which reaches more remote parts. So that in practice one dose of a radio-active substance may, even in the same organ or tissue, produce a destructive effect at close quarters and the very reverse effect at a slightly greater distance. These observations hold good for the cells of malignant growths even more than for the normal tissues; but, as with the latter, different types of growth react in varying degrees to the $\beta$ radiations.

The $\gamma$ radiations cause reactions in the living tissues which are distinctly different from those of the $\beta$ radiations. The $\gamma$-rays are less antiseptic, and less fibrous tissue is formed after recovery from the exposure; if a burn of the surface has been produced by too intense a radiation, the resulting scar is much more likely to be the seat of disfiguring telangiectasis than when the same accident follows $\beta$ radiation. In actual practice to-day, $\beta$ an $\gamma$ radiations are used together. The germicidal properties of the $\beta$-rays are valuable for infective processes, such as tuberculous and other ulcers and the secondary infections so common in malignant disease. Both kinds act upon malignant disease, though it is not at all easy to predict what the action will be. Sometimes it is destructive, sometimes it is even stimulative, sometimes it is negligible either way. Many observers believe that there is a *selective action upon the cells of malignant tumours*, which results in the deaths of those cells without fatal results upon surrounding normal cells. Indeed, the theory of the treatment of malignant diseases by radio-active substances depends upon the existence of such a selective action. There is a good deal of evidence to support this view, but it is not yet established, and for the present it should be accepted with caution, if at all.

